# High-resolution mucociliary transport measurement in live excised large animal trachea using synchrotron X-ray imaging

**DOI:** 10.1186/s12931-017-0573-2

**Published:** 2017-05-16

**Authors:** Martin Donnelley, Kaye S. Morgan, Maged Awadalla, Nigel R. Farrow, Chris Hall, David W. Parsons

**Affiliations:** 1 Robinson Research Institute, University of Adelaide, Adelaide, SA 5001 Australia; 2 Respiratory and Sleep Medicine, Women’s and Children’s Hospital, 72 King William Road, North Adelaide, SA 5006 Australia; 3 Adelaide Medical School, University of Adelaide, Adelaide, SA 5001 Australia; 4 School of Physics and Astronomy, Monash University, Clayton, Vic 3800 Australia; 5 Institute for Advanced Study, Technische Universität München, Munich, Germany; 6 Imaging and Medical Beamline, Australian Synchrotron, Clayton, Vic 3800 Australia

**Keywords:** Mucociliary transport, Trachea, Particle tracking, Cystic fibrosis, Phase contrast, X-ray imaging, Synchrotron

## Abstract

**Background:**

The Australian Synchrotron Imaging and Medical Beamline (IMBL) was designed as the world’s widest synchrotron X-ray beam, enabling both clinical imaging and therapeutic applications for humans as well as the imaging of large animal models. Our group is developing methods for imaging the airways of newly developed CF animal models that display human-like lung disease, such as the CF pig, and we expect that the IMBL can be utilised to image airways in animals of this size.

**Methods:**

This study utilised samples of excised tracheal tissue to assess the feasibility, logistics and protocols required for airway imaging in large animal models such as pigs and sheep at the IMBL. We designed an image processing algorithm to automatically track and quantify the tracheal mucociliary transport (MCT) behaviour of 103 μm diameter high refractive index (HRI) glass bead marker particles deposited onto the surface of freshly-excised normal sheep and pig tracheae, and assessed the effects of airway rehydrating aerosols.

**Results:**

We successfully accessed and used scavenged tracheal tissue, identified the minimum bead size that is visible using our chosen imaging setup, verified that MCT could be visualised, and that our automated tracking algorithm could quantify particle motion. The imaging sequences show particles propelled by cilia, against gravity, up the airway surface, within a well-defined range of clearance speeds and with examples of ‘clumping’ behaviour that is consistent with the in vivo capture and mucus-driven transport of particles.

**Conclusion:**

This study demonstrated that the wide beam at the IMBL is suitable for imaging MCT in ex vivo tissue samples. We are now transitioning to in vivo imaging of MCT in live pigs, utilising higher X-ray energies and shorter exposures to minimise motion blur.

**Electronic supplementary material:**

The online version of this article (doi:10.1186/s12931-017-0573-2) contains supplementary material, which is available to authorized users.

## Background

Cystic fibrosis (CF) lung disease is caused by an improperly functioning ion channel in the airway epithelium and results in a reduction in the depth of the airway surface liquid (ASL) layer and impaired mucociliary transport (MCT). MCT is the mechanism by which tiny hair-like structures called cilia move inhaled particles along the airway surfaces and out of the lungs. In CF, the airway dehydration and production of thick sticky mucus result in a cycle of inflammation and infection that ultimately leads to progressive lung disease and early death from lung failure [1].

We have developed an airway gene transfer technique that inserts the corrective gene (CFTR) into defective airway cells of CFTR-null mice, with the resulting CFTR ion-channel correction lasting for at least 12 months [2]. Although we can electrically measure the ionic changes produced by our gene therapy using the established transepithelial potential difference measurement technique, there are currently no methods to rapidly and accurately quantify small corrections in the dysfunctional mucociliary activity or any improvements in airway health in a live animal, after a potential treatment is performed. The ability to detect beneficial effects produced by novel therapies in live CF airways soon after treatment would substantially advance research into new genetic and pharmaceutical CF treatments.

MCT has typically been quantified by measuring the bulk clearance of radioactive or fluorescent particles deposited in the airways [3]. To rapidly determine the efficacy of genetic and pharmaceutical therapies for CF we have developed MCT monitoring methods in live anaesthetised normal and CFTR-null mice to measure the transport rate and behaviour of individual deposited 10-30 μm diameter high refractive index (HRI) glass bead marker particles using propagation-based synchrotron phase contrast X-ray imaging (PCXI) [4]. Compared to conventional sources, a synchrotron can produce X-rays that are much brighter, more spatially coherent and closer to monochromatic, enabling PCXI setups able to utilise X-ray refraction (in addition to absorption) to achieve high spatial and temporal resolution, and excellent airway and lung soft tissue contrast. Nuclear imaging techniques, fluoroscopy and CT imaging systems are not able to resolve particles of this size. These synchrotron X-ray MCT imaging methods have been developed in mice over more than 8 years at the SPring-8 synchrotron in Japan, and we have recently begun to transition them to the Imaging and Medical Beamline (IMBL) at the Australian Synchrotron [5].

However, CFTR-null mice are a limited model because they do not exhibit human-like lung pathophysiology [6], and although the nasal airways of these mice do exhibit the CF pathophysiology, there is evidence that nasal MCT is not altered in CFTR-null mice [4, 7]. Furthermore, since it is designed for large animal imaging, the IMBL beam characteristics are not well suited to resolving the 10–30 μm particles normally used for MCT measurement in a ~1 mm diameter mouse airway.

Our group is interested in using newly developed large-animal CF animal models, such as the CF pig, which display human-like lung disease and have human-sized lungs [8]. In future imaging experiments we plan to test the effectiveness of our gene therapy treatment in live CF animals at the IMBL, to rapidly and non-invasively determine the effectiveness of our gene therapy protocols via assessment of MCT-rate changes. The IMBL is primarily designed for performing propagation-based PCXI due to its high x-ray flux and high coherence, partly brought about by a large source-to-sample distance (greater than 137 m in the satellite building hutch). The beamline setup produces an X-ray beam of up to 400 mm × 40 mm (W × H) at the sample position, making the imaging of larger animals such as sheep, pigs, and potentially humans, possible in the future.

A recent article has detailed the importance and feasibility of MCT analyses for CF, and revealed the differences between CF and normal airways, both at baseline and under pharmaceutical manipulation [9]. That study used a standard human clinical CT setup combined with large (350 μm diameter x 25 μm thickness) tantalum discs as MCT markers that were insufflated into the airways using a puff of air. CT scans were acquired every 15 s for 10 minutes to track micro-disc motion, and used a minimum detection velocity of 1 mm/min. They showed that a similar proportion of micro-discs cleared the CF and non-CF airways over the 10-minute period, and that in both groups the discs migrated toward the ventral tracheal surface. Impaired clearance in the CF group was only apparent when a methacholine challenge was applied to both. Importantly, they noted that MCT assays could be used to report therapeutic efficacy.

The aim of the present study was to report the feasibility of using PCXI at the IMBL to quantify MCT in excised normal large animal airways using HRI glass marker particles. We used freshly excised normal tracheal tissue from sheep and pigs to remove the imaging complexity that would be produced by surrounding body tissues. We aimed to use our ex vivo imaging system to determine the range of MCT rates, whether there are preferential patterns of MCT flow within the trachea, and to demonstrate the effect of common clinical rehydrating therapies for CF airways that are known to alter MCT [10]. Importantly, we aimed to obtain the baseline MCT data from normal large animal trachea as well as permit the establishment of the necessary logistics and IMBL needs and protocols for airway MCT imaging. This study was essential for our continued efforts to establish imaging and technical infrastructure required for the transition to in vivo trials in large animal models such as normal and CF pigs, in order to validate the effects of genetic and pharmaceutical therapies.

## Methods

The Imaging and Medical Beamline at the Australian Synchrotron was used for all imaging experiments, and the data was collected over two beamtime allocations.

### Beamline setup

The storage ring operated at 3 GeV and a ring current of 200 mA for both experiments. The RMS electron beam size in the straight sections was 320 × 16 μm [11], and corresponded to a Gaussian FWHM of 754 × 38 μm in the horizontal and vertical directions, respectively. The IMBL X-ray beam was produced by a superconducting multi-pole wiggler functioning at a field strength of 3.0 T. The required energy of 30 keV was selected using a dual silicon Laue crystal monochromator, and the source-to-monochromator distance was 16.15 m.

All imaging was performed in the IMBL satellite building experimental hutch 3B at a distance of ~136 m from the storage ring [12], using the previously established setup shown in Murrie et al. [13]. A sample to detector distance of 2.5 m was used, and images with an effective pixel size of 16 μm (field of view of ~ 16.6 mm × 14 mm) were captured using the Ruby detector [14]. This was the smallest field of view of which the detector was capable, and was selected in order to visualise most of the width of the ~15–20 mm diameter tracheae at high magnification.

X-ray beam uniformity is to a certain extent governed by the power load on the monochromator. For the first experiment the power of the input spectrum was reduced using copper filters, resulting in a lower flux and uniform illumination. Exposure times of 1 s were required to use most of the dynamic range (16-bits) of the PCO.edge sensor (PCO imaging, Germany). In the second experiment the copper filters were removed, increasing the available flux and enabling an exposure length of 250 ms to be used, at the expense of a slight degradation in the quality of the beam shape.

### MCT tracking particle testing

Prior to the excised tissue experiments, the visibility of a selection of differently-sized HRI glass beads (Corpuscular, NY, USA) was assessed using the chosen imaging parameters. Minute quantities of HRI beads with diameters of 52, 78 and 103 μm were placed on Kapton tape and imaged.

### Excised tissue preparation

The first experiment utilised five freshly-excised normal adult sheep tracheae and the second utilised three freshly-excised normal adult pig tracheae (Camborough 29, ~250 kg female); all samples were recovered from animals off-site from the synchrotron. Animals were sedated and then humanely killed by sodium pentobarbital overdose, with the trachea rapidly excised immediately after death. Care was taken to minimise the amount of blood that entered the tracheae. After excision each trachea was washed in a solution of 20 mM Krebs-HEPES Buffer at pH 7.4, and stored for transport to the IMBL in a fresh container of the same buffer solution at ~4 °C.

At the IMBL each trachea was cut into ~20 mm long segments (typically resulting in 3–5 segments per trachea, containing ~3 cartilage rings each, with each segment slightly taller than the imaging field of view). Prior to imaging, the tracheal segment was pinned out vertically onto the base of a custom-made polycarbonate trachea chamber on well-moistened gauze, then slowly warmed to and maintained at physiological temperature (~37 °C). The segments were normally mounted with the cranial end at the top, so that the predominant direction of MCT was upwards, against gravity. In the sheep experiment two segments were mounted “upside down” to verify that MCT occurred in the expected direction (downwards in this “upside down” case). In the pig experiment four segments of conducting airway tissue from, at and below the carina were also examined.

Samples were typically imaged within 8 h of excision except for one during the sheep experiment that was deliberately imaged ~24 h after excision to assess whether residual MCT activity was present after a prolonged period in buffer at 4 °C.

### Imaging

Each tracheal segment was placed on the hutch X-Y stage with temperature maintained at ~37 °C, and oriented such that images were captured from the dorsal/ventral (anterior/posterior) direction. In the pig studies images of the trachea were captured prior to HRI particle delivery, to be used for background subtraction. A small quantity of 103 μm diameter HRI glass particles were then delivered to the caudal end of the tracheal segment using a mouse Dry Powder Insufflator™ (PennCentury, PA). For the sheep studies the particles were placed on the ventral tracheal surface (i.e. the side opposite the trachealis muscle and oesophagus where MCT was expected to be maximal [9]), and for the pig studies on the dorsal tracheal surface (to detect any dorsal to ventral particle motion).

**Fig. 1:**
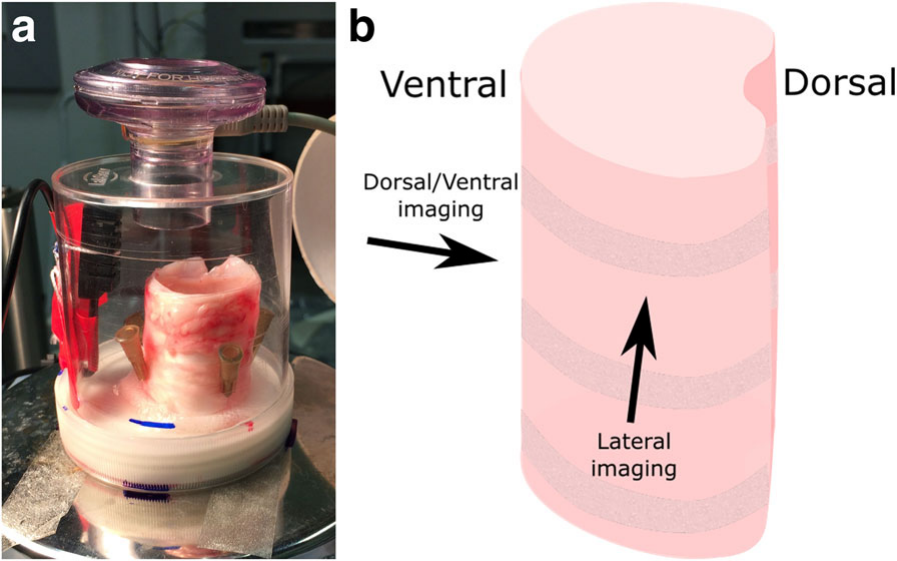
Tracheal imaging setup. **a** A pig tracheal segment in the ex vivo sample chamber. The segment is pinned to a wax base using 25 Ga syringe needles to prevent movement. The Aeroneb nebuliser is shown inserted into the top of the sample chamber, and was used to deliver aerosolised isotonic or hypertonic saline to the tissue. A temperature and humidity sensor is visible on the left side of the container. The entire chamber was maintained at 37 °C, but relied on passive humidification from the wet gauze in the chamber. The same setup was used for imaging the sheep tracheal segments. **b** The tracheal tissue orientation, with imaging (x-ray beam) directions corresponding to those used in Figs. 3 and 4

Ten minutes of baseline imaging was performed. For some samples aerosolised 7% hypertonic saline, 0.9% isotonic saline, or 9% mannitol were delivered to the tracheal segment using an Aeroneb vibrating mesh nebuliser (Aerogen, Ireland) inserted into the top of the chamber, to test the effects of treatments known to alter airway surface hydration. Aerosol was delivered for 30 s at 50% duty cycle (equivalent to 15 s total aerosol generation, and ~50 μl liquid). Post-treatment imaging continued for 10 min to evaluate the duration of active MCT clearance mechanisms against the same sample baseline, and control tracheal samples. An example of a tracheal segment prepared for imaging is shown in Fig. 1.

Hoegger et al. showed that deposited particles preferentially migrated to the ventral tracheal surface [9, 15], so to test whether this effect was detectable in our samples some segments were rotated axially 90° after the first imaging run was completed. An additional sample of beads was deposited in the same manner, but this time onto the lateral tracheal wall. A second imaging run, including aerosol delivery, was then performed in a lateral direction to compare the MCT and spatial pattern of particle movement beginning on a ventral or a lateral wall of the trachea.

### Post-experimental analyses

Custom image analysis software (written by MD and described in Appendix A) was used to measure and compare particle MCT rates by identifying particles and tracking their motion throughout the image sequences.

To validate the automated tracking algorithm the first frame of each imaging run was manually analysed to count the number of features incorrectly identified as particles (false positives) and the number of moving particles that were not correctly identified or tracked (false negatives). These values were used along with the number of detected particles (positives) to determine the sensitivity of the algorithm.

Where appropriate, statistical analyses were performed using GraphPad Prism 7. Data were tested for normality, statistical significance was set at *p* = 0.05 and power = 0.80, and a one-way ANOVA and Bonferroni post-hoc test were used for multiple comparisons. MCT rates are presented as mean plus standard deviation.

## Results

**Fig. 2:**
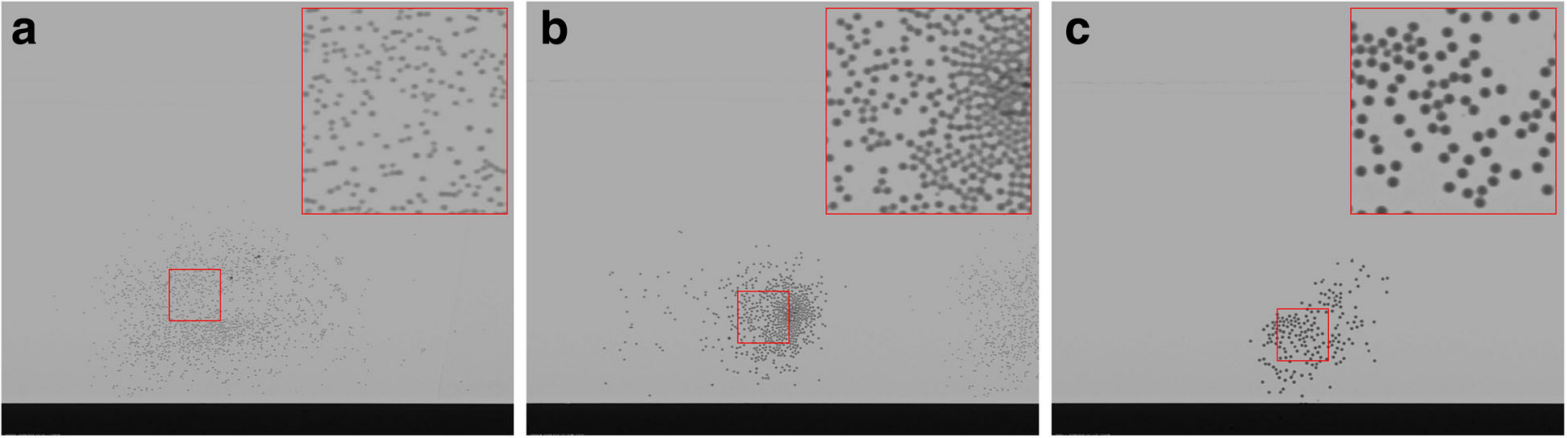
X-ray images of differently-sized MCT tracking particles. Images of (**a**) 52 μm, (**b**) 78 μm and (**c**) 103 μm HRI glass beads on Kapton tape, taken at 30 keV with the field of view set to ~ 16.6 mm x 14 mm to image the full width of the tracheal segments. The contrast is primarily due to absorption. The insets show a 4x magnification of the beads. The brightness and contrast of the three images have been altered in the same way to improve particle visualisation for publication and allow visual comparisons. The 103 μm beads were chosen for the ex vivo experiments. Scale bar 2 mm

At the chosen magnification all three bead sizes were visible on the Kapton tape (see Fig. 2). However, to ensure the beads were visible and could be reliably tracked in the excised tissue segments, and in future live animal studies where overlying tissue and respiratory motion typically complicates image analysis, the 103 μm sized beads were chosen.

A breakdown of the number of samples in each group is shown in Table 1.

**Table 1:** The number of imaging runs in which MCT tracking particles were successfully tracked in each group

Imaging direction	Treatment	Sheep (*n* = 5)	Pigs (*n* = 3)
Dorsal/Ventral	Baseline	14	15
	Hypertonic	1	7
	Isotonic	0	6
	Mannitol	0	3
Lateral	Baseline	14	10
	Hypertonic	3	2
	Isotonic	4	3
	Mannitol	0	4
Other	Upside down	2	0
	At 24 h	1	0
	Lung	0	4
		39	51

Note that multiple runs (i.e. baseline followed by aerosol treatment) were performed in some segments

**Fig. 3:**
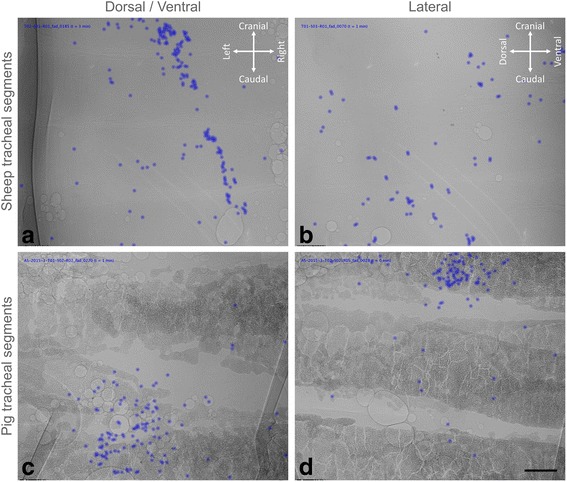
MCT tracking particles in sheep and pig tracheae. Example high magnification images of tracked 103 μm HRI glass MCT marker particles in segments of excised sheep trachea (**a** and **b**) and pig trachea (**c** and **d**), from a dorsal/ventral view (**a** and **c**) and lateral view (**b** and **d**). For the lateral images, the dorsal tracheal surface is to the left and the ventral tracheal surface to the right. The particles successfully detected and tracked by our automated algorithm are marked with blue dots. The differences in cartilage texture and density between the sheep and pigs are clearly visible. In panel (**c**) the edges of the plastic hubs of two syringe needles used to pin the segment in place can be seen on the left and the right side of the image. Additional file 1: Video S1 and Additional file 2: Video S2 correspond to panels (**a**) and (**c**), respectively. Scale bar 2 mm



The MCT particle tracking algorithm was able to successfully detect and track particle motion in all of the sheep tracheal segments. An example of the tracked particles is shown in Fig. 3 and Additional file 1: Video S1 and Additional file 2: Video S2. Data from three of the pig tracheal segments (Baseline + Iso, Baseline + Mannitol, and Lateral Baseline) was excluded due to tissue motion over the imaging period that made the background subtraction algorithm (described in Appendix A1.2) fail. A total of 1.37 million particles (average 35,035 per imaging run, or 234 per frame) and 3.8 million particles (average 73,089 per imaging run, or 122 per frame) were detected and tracked by the algorithm for the sheep and pig experiments, respectively. The sensitivity was 96% in the sheep experiment and 87% in the pig experiment. Particle identification was more challenging in the pig tracheal segments than the sheep segments because the pig cartilage rings were much denser (and therefore more strongly absorbing at the chosen energy of 30 keV) as well as more textured (at times having features of a similar appearance to the MCT tracking particles), and there was little clear space between the cartilage rings (see Fig. 3). However, apart from in the excluded image sequences in which the segments moved during the imaging period, there were few false positives at the particle identification step. When false positives did occur they were often caused by bubbles on the airway surface. These were not present in many tracheal segments, but there were some in the airway from deeper in the lung (see Additional file 3: Video S3). Due to their infrequent occurrence relative to the true positives, and the fact that they did not move substantially during tracking, these false positives did not skew the results.

**Additional file 1:** Video S1. Were assembled from the processed images to show the variability in particulate behaviour on the tracheal airway surfaces. The movie frame rate was set to 5x normal speed. Additional file Videos can be played using the free VLC Media Player (available at http://www.videolan.org/vlc/). shows MCT tracking particles in the segment of sheep trachea shown in Fig. 3a, during a baseline (no aerosol) run, taken from a dorsal/ventral view. The majority of particles are correctly tracked, but occasional un-detected particles are visible. (MP4 21840 kb)




**Additional file 2:** Video S2. Were assembled from the processed images to show the variability in particulate behaviour on the tracheal airway surfaces. The movie frame rate was set to 5x normal speed. Additional file Videos can be played using the free VLC Media Player (available at http://www.videolan.org/vlc/). shows tracked particles in a baseline run from a dorsal/ventral view in the segment of pig trachea shown in Fig. 3c. The direction of transport is clearly different to the sheep trachea. (MP4 10903 kb)




**Additional file 3:** Video S3. Were assembled from the processed images to show the variability in particulate behaviour on the tracheal airway surfaces. The movie frame rate was set to 5x normal speed. Additional file Videos can be played using the free VLC Media Player (available at http://www.videolan.org/vlc/). shows MCT particle motion at the segment of airway containing the pig carina, shown in Fig. 7. A number of false positives were present in this run, due to bubbles on the airway surface. (MP4 21962 kb)


When particles aggregated very closely the particle detection step (described in Appendix A) was unable to accurately separate them into individual particles (producing false negatives). Accordingly, when beads were isolated they were more accurately identified and tracked than when aggregated into groups. This happened more frequently in the pig tracheal segments, but again had little effect on the results because all particles in the aggregates normally moved at similar speed. Often particles were not tracked for the entire image sequence, with the algorithm occasionally losing a particle for a frame or two before detecting it again in subsequent frames. In the sheep experiment there were some beads that moved too fast to be accurately detected by the tracking code (described in Appendix A1.4). Since we used a 15 pixel maximum displacement to ensure tracking was successful, particles moving faster than 6 mm/min could not be tracked by this algorithm (see Additional file 1: Video S1). This may have reduced the measured mean MCT rate in the sheep experiment, and was one of the primary reasons we increased the frame rate for the second (pig trachea) experiment. For the pig tracheal segments the theoretical maximum MCT rate that could be measured by the tracking algorithm was 24 mm/min.

Particle MCT was visible in all tracheal segments examined, including the sheep segment that was deliberately stored in chilled buffer solution for ~24 h after excision. In the normally-prepared (caudal end upwards) samples beads moved in the direction of preferential MCT, primarily up the tracheal wall when oriented against gravity, although interestingly they rarely moved directly up the trachea at an angle of zero degrees. In the two sheep tracheal segments that were mounted “upside down” MCT also proceeded downwards, as expected. Beads tended to organise and aggregate into long strings, and appeared to move in favoured MCT tracts. In the pig samples the beads typically moved circumferentially towards the ventral tracheal surface.

The mean instantaneous baseline MCT rate was 1.62 ± 1.22 mm/min for the sheep, and 1.32 ± 1.19 mm/min for the pigs. Following treatment with aerosolised hypertonic saline the average MCT rate over the 10-min period rose to 2.50 ± 1.19 mm/min for the sheep, and 1.75 ± 1.59 mm/min for the pigs; clearly individual rates would have been higher at some time points. For the sheep tracheal segment imaged upside down the instantaneous MCT rate was 1.45 ± 0.81 mm/min, and for the segment imaged 24 h after excision the MCT rate was 0.34 ± .0.3 mm/min. In all cases the measured mean MCT rates were lower than those previously reported in humans; 5.7 ± 2.6 mm/min [16] and 4.1 mm/min [16, 17] as well as in sheep; 8.2 ± 1.9 mm/min [18] live pigs; 6.9 ± 0.7 mm/min [15] and excised pig trachea; 2.3 ± 0.3 mm/min [19]. Although we did see many particles moving at similar rates to these, the presence of large numbers of very slow moving particles lowered the average rate. From our pilot SPring-8 studies in mice we suspect that the choice of MCT tracking particles (size and surface characteristics) may also play a role in the particle transport rate, and may also be responsible for some of the difference in our reported MCT rates.

**Fig. 4:**
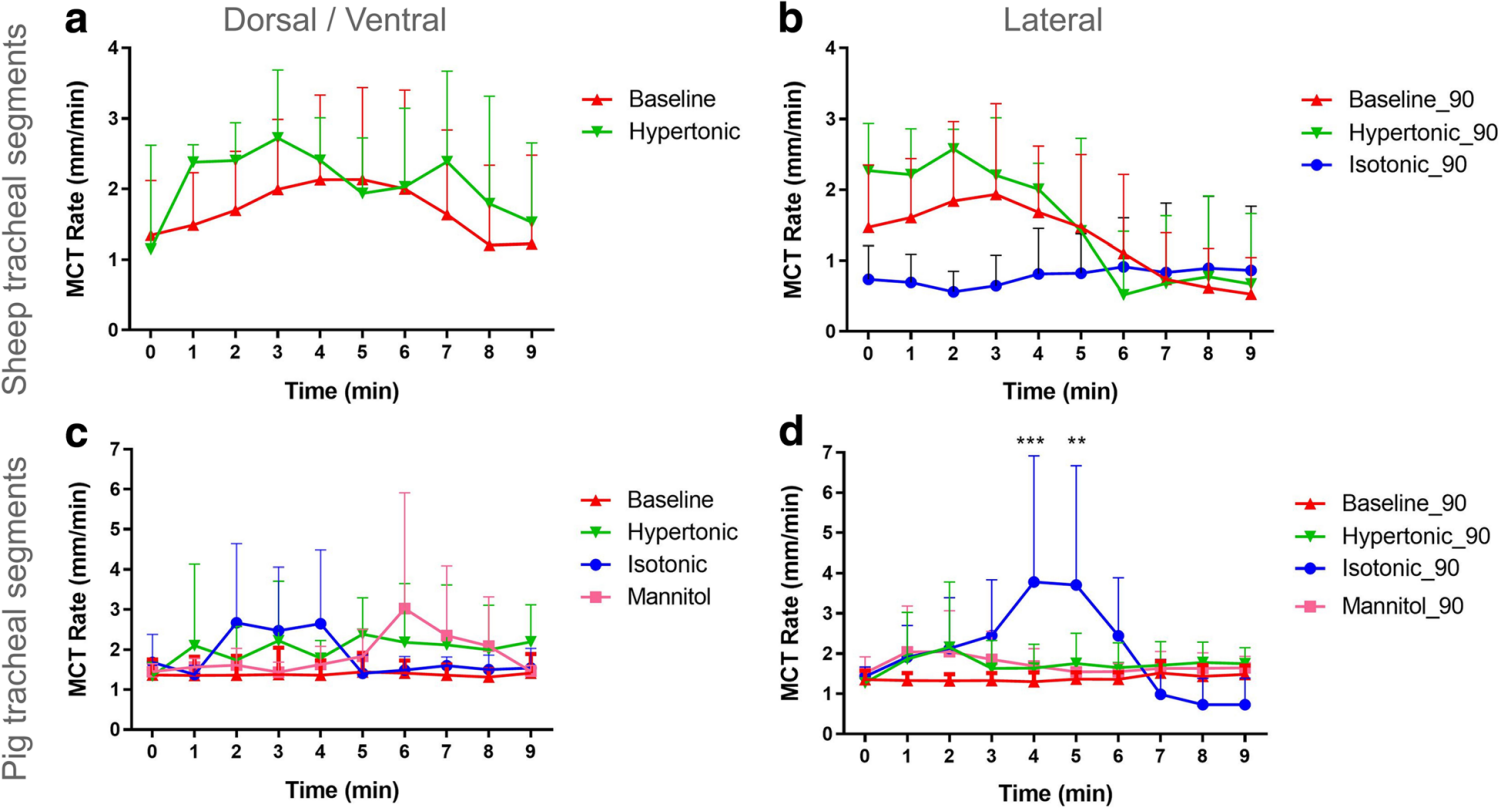
Response of pig and sheep MCT rate to treatment. Average MCT rate over time in response to treatment in (**a**) sheep dorsal/ventral, (**b**) sheep lateral, (**c**) pigs dorsal/ventral, and (**d**) pigs lateral (**p < 0.01, ***p < 0.001, ANOVA vs baseline, mean + SD, *n* = 3)

The change in MCT rate in response to treatment over the 10-min imaging period is shown in Fig. 4. The data are clearer for the lateral view, and shows a transient increase in MCT rate following aerosol delivery that rapidly returned towards normal. This effect was not statistically significant, likely because of the small number of aerosol treated segments, particularly in the sheep experiment, as well as the wide variability in the MCT rate of the individual beads.

**Fig. 5:**
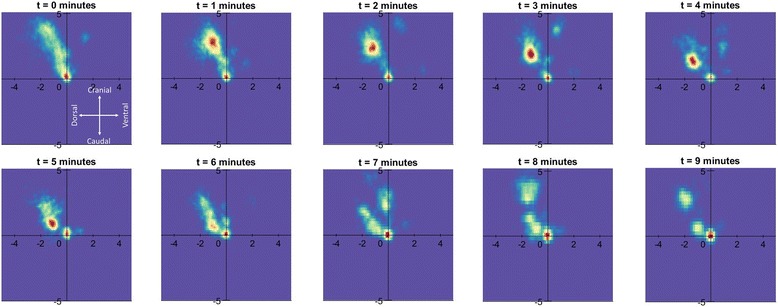
Lateral heat-map for MCT behaviour in sheep trachea. Two dimensional heat-map showing the effect of aerosolised hypertonic on the predominant MCT particle velocity across the three relevant sheep tracheal segments (*n* = 2 sheep) over the 10-minute imaging period. The heat-map orientation is the same as Fig. 3b and d; the dorsal tracheal surface is to the left and the ventral tracheal surface to the *right*. Stationary particles are at the origin, slow moving particles are close to the axis origin, and faster moving particles are further away, with the angular position representing their direction of motion. *Red* indicates more particles than *yellow* or *blue*. The heat maps indicate that the bulk of the particle motion is in a cranial direction, with a bias towards the dorsal tracheal surface (the average angle of particle transport was -14.3° from vertical)

**Fig. 6:**
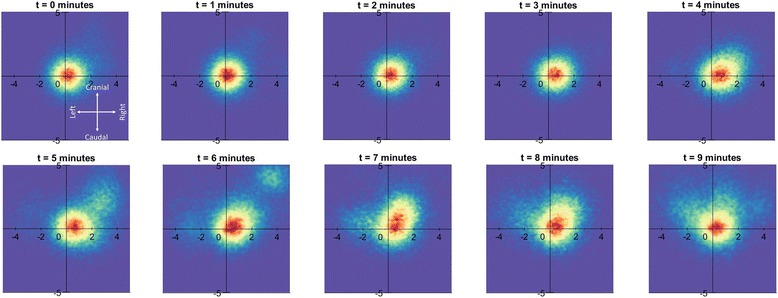
Dorsal/ventral heat-map for MCT behaviour in pig trachea. Two dimensional heat-map showing the effect of aerosolised hypertonic on MCT particle velocity in the seven pig tracheal segments (*n* = 3 pigs) over the 10-minute imaging period. The heat-map orientation is the same as Fig. 3a and c. With treatment the hot spot shifts slightly right, indicating that the bulk of the particle movement is laterally towards the right lateral wall (the average angle of particle transport was +28.3° from vertical)

To examine MCT directionality the magnitude and direction of the instantaneous velocity of each tracked particle was plotted on a two-dimensional heat-map (See examples in Figs. 5 and 6). For the baseline runs (data not shown) the hotspot remains close to the origin as the bulk of the particles are moving slowly. Note that the t = 0 image corresponds with the first 15 s of aerosol delivery, so some particles have already begun to move faster than at baseline, also suggesting that the aerosol begins to act very rapidly on some particles. The MCT rate of many particles transiently increases with treatment, but returns toward normal at the later time points.

**Fig. 7:**
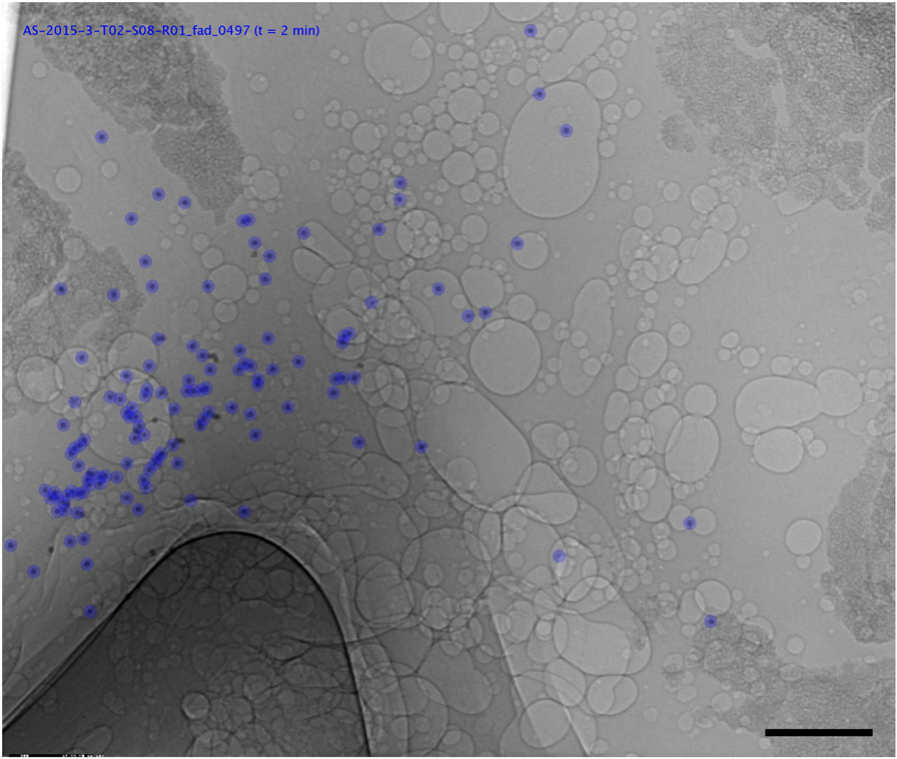
Particle tracking at the pig carina. Particles could also be successfully tracked at the pig carina. In this sample the presence of the bubbles on the airway surface increased the false positive detection rate. Additional file 3: Video S3 shows the tracking results for this sample. Scale bar 2 mm

For one of the pigs we also excised tissue from deeper within the lung, including the carina, and verified that particle transport could be tracked there in the same manner (See Fig. 7 and Additional file 3: Video S3).

## Discussion

The predominant direction of MCT appeared different between species, being along the length of the trachea in the sheep, and more circumferentially in the pigs (see Additional file 1: Video S1 and Additional file 2: Video S2). This may have been because in the sheep the particles were deposited on the ventral wall – the portion of the trachea that Hoegger et al. showed particles migrate towards [9, 15] – whereas in the pigs the particles were deposited on the dorsal wall, and therefore had a more axial motion to transport towards the ventral wall (See Additional file 1: Video S1 and Additional file 2: Video S2). Further studies must be performed to confirm this speculation.

At SPring-8, we have exploited synchrotron PCXI on a highly coherent source (BL20XU and BL20B2) for live small-animal imaging (mice and rats) where high flux is critical to obtain very short exposure times (<50 ms) to avoid motion blurring in animals with high respiratory rates. High spatial coherence is necessary for PCXI, however the experiments we describe here, with a larger field of view and strong absorption contrast from the beads, demonstrate that the reduced spatial coherence at the Australian Synchrotron’s IMBL is sufficient for our methods, making these experiments in large airways viable at the IMBL. We expect that when imaging a ~20 kg piglet (i.e. 8–9 weeks old) the additional neck tissue surrounding the trachea will require the use of a higher energy (e.g. 60 keV) with an exposure time of around 100 ms to maintain motion-free images during respiration. However, based on the results from this study, we predict that in vivo MCT measurements in a live pig trachea are feasible.

These clear pilot data has shown the exciting potential of the IMBL for performing live large animal imaging when relevant large normal and CF animal models are appropriate and available. These studies did have a number of limitations, including the rudimentary maintenance of humidification (closed imaging vessel held at 37 °C) and the variability in the time between excision and imaging for each segment. There was also an absence of reciprocating airflow within the tracheal segment that would mimic the normal lung airflow conditions during breathing; such airflow conditions are known to release airway nucleotides that can influence ASL depth and MCT rate [20]. However, these experiments were designed as ex vivo studies using scavenged tracheal tissue, to trial expected imaging, biological, and logistical parameters with sufficient experimental flexibility to enable imaging optimisation without the complications of strict radiation dose control and live-animal handling restrictions. Provided physiological support is utilised to maintain adequate tissue hydration and temperature, it would be possible to use this ex vivo setup to image multiple particles in various locations within large animal airway segments over long time periods (e.g. multiple hours), and to examine the effects of mucus reducing agents or other pharmaceuticals on MCT function.

When extended to in vivo tracheal imaging using higher X-ray energies for improved penetrating power (and minimise the effects of overlying tissue and cartilage) we expect to be able to track multiple small particles, perform repeat imaging, and image for long periods; a protocol that will be useful for studying the effects of long-acting pharmaceuticals or genetic therapies on MCT. Although imaging of the deeper airway was possible in this study, we expect that in live imaging experiments the greater lung motion in the deeper lung regions would make accurate measurements in this region much more challenging. The primary benefits of using this type of two-dimensional imaging setup compared to CT (e.g. as used by Hoegger et al. [9, 15]) is that it can be used to track treatment effects for long periods with reduced radiation exposure, it can track multiple individual particles with high spatial and temporal resolution and thereby examine clearance heterogeneity and particulate grouping behaviour within the trachea. This may be particularly informative in CF models where some portions of the airway have dysfunctional clearance while other areas retain essentially normal clearance [21]. Future in vivo studies will deal with essential aspects of in vivo experimental design, and with reductions in radiation dose we expect that repeated-measures studies that allow for the tracking of treatment effects will be possible and allow assessment of MCT over longer periods (as already successfully performed in mice at the SPring-8 Synchrotron in Japan [22]).

## Conclusion

This experiment demonstrated that the IMBL X-ray beam is suitable for imaging MCT in ex vivo tissue samples from large animals. Our findings indicate that with increased flux and reductions in exposure length the measurement of MCT behaviour via PCXI imaging will be possible in future live-imaging studies of intact large animals such as sheep and pigs.

### Additional files

Additional file 4: Figure S1. Example of the background subtraction images. Example background subtraction images corresponding to Fig. 3a and c, respectively. Scale bar 2 mm. (JPG 1321 kb)

## Appendix 1: Particle tracking algorithm

The MCT rate of the marker particles in each tracheal segment was determined using an automated analysis workflow consisting of image pre-processing, background subtraction, particle identification, particle tracking, and visualisation. All were performed using custom software written in Matlab (R2016b, The Mathworks, Natick, MA, USA).

### A1. 1. Image pre-processing

Flat-field and dark-current correction were performed to minimise the effects of dust and scratches on the scintillator, and to minimise inhomogeneities in the X-ray beam intensity.

### A1. 2. Background subtraction

In the pig study there was too large a shift in the tissue sample position as a result of the particle delivery procedure for the background images acquired prior to particle delivery to be useful for background subtraction. Instead, we loaded ~60 equi-spaced images from the imaging run and extracted the maximum pixel value for each pixel position through the stack to create a composite image (See Additional file 4: Figure S1). This process chose the brighter background over the dark beads, and resulted in a background image that contained no particles unless they were stationary for the entire imaging run. Foreground images containing only the moving particles were then created by subtracting the background image from every image in the run.

### A1. 3. Particle identification

To allow changes in particle behaviour in response to treatment to be assessed over time, every 10-minute imaging block was split so that particle identification and tracking was performed in ten 15-s blocks spaced 1 minute apart. Circular particles were identified in each image in a block using the circular Hough transform. True HRI marker particles were then selected based on particle radius, with a range of 7.5 to 9.5 pixels (diameter 97 to 123 μm) empirically chosen to ensure the algorithm identified most particles. The generous upper limit allows for a small amount of motion blur that increases the apparent diameter of the beads. The location of every particle and the frame number in which it was identified were stored in a Matlab data structure.

### A1. 4. Particle tracking

Once every particle in each frame of a 15-s block was identified, the particle locations were linked into trajectories using a particle tracking algorithm developed by Georgetown University [23]. Their algorithm examines the positions of all *n* particles at time *t(i)*, and the *m* possible new positions at time *t(i + 1)*, and considers all possible combinations of the *n* old positions with the *m* new positions, choosing the combination that results in the minimal total squared displacement. In their algorithm the trajectories are controlled by a displacement coefficient that represents the maximum distance a particle can move between two frames. This parameter should be set lower than the mean spacing between the particles, and when it increases the run-time also increases significantly due to the much larger number of possible tracking combinations that must be evaluated. For this study the maximum displacement parameter was set to 15 pixels (~100 μm), which was the largest possible value that allowed the tracking code to run for all the tracheal samples. The coordinates of the tracked particles and their corresponding frame numbers were stored into a new data structure. Particles that were tracked for less than four consecutive frames were excluded.

The distance that each particle moved between frames was calculated in pixels and converted to millimetres, based on the size of the field of view. The MCT rate of each particle was then calculated (in mm/min) based on the time between analysis frames. The distance that every tracked particle moved between every frame was reported as the *instantaneous* particle MCT rate. All the instantaneous MCT rates for each particle were then averaged to produce an average MCT rate for each particle, which was reported as the *average* particle MCT rate.

### A1. 5. Visualisation

The location of every tracked particle in each of the analysed frames was marked (see Fig. 3). Particles for which there was no reliable tracking data were not marked, even if they were correctly identified by the code described in Section A1.3.

Instantaneous tracking particle velocity histograms were generated based on the magnitude and direction of particle motion, and presented as a two dimensional heat map [24]. Data was either separated and shown for each imaging run (not shown), or data for each group was pooled together and separated into time blocks (Figs. 5 and 6).
