# Supplementary figures and images for: High-resolution mucociliary transport measurement in live excised large animal trachea using synchrotron X-ray imaging
Source: Respir Res. 2017 May 16;18:95. doi: 10.1186/s12931-017-0573-2 (PMC5434541; doi:10.1186/s12931-017-0573-2)

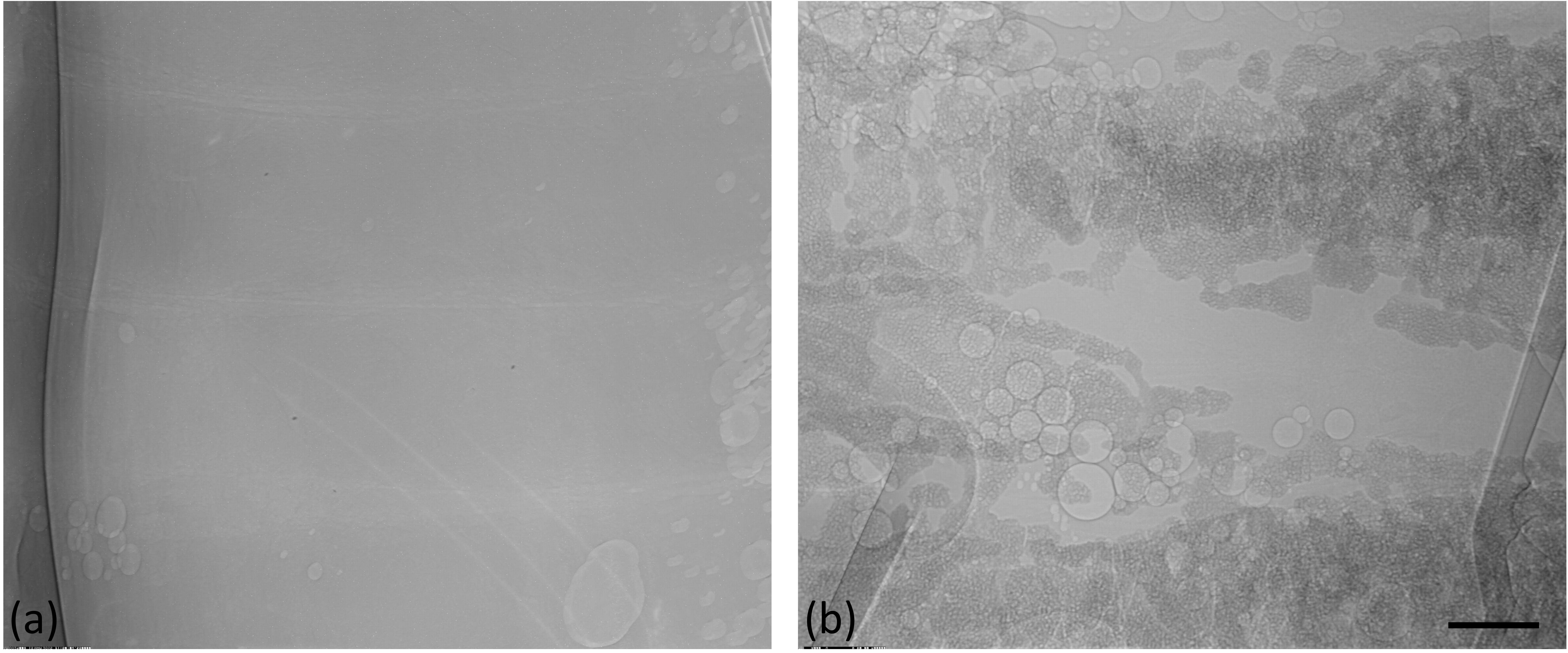

Supplement: Supplementary file 4 — Example of the background subtraction images. Example background subtraction images corresponding to Fig. 3a and c, respectively. Scale bar 2 mm. (JPG 1321 kb) [file 12931_2017_573_MOESM4_ESM.jpg]
